# A large open access dataset of brain metastasis 3D segmentations on MRI with clinical and imaging information

**DOI:** 10.1038/s41597-024-03021-9

**Published:** 2024-02-29

**Authors:** Divya Ramakrishnan, Leon Jekel, Saahil Chadha, Anastasia Janas, Harrison Moy, Nazanin Maleki, Matthew Sala, Manpreet Kaur, Gabriel Cassinelli Petersen, Sara Merkaj, Marc von Reppert, Ujjwal Baid, Spyridon Bakas, Claudia Kirsch, Melissa Davis, Khaled Bousabarah, Wolfgang Holler, MingDe Lin, Malte Westerhoff, Sanjay Aneja, Fatima Memon, Mariam S. Aboian

**Affiliations:** 1 Yale School of Medicine, Department of Radiology and Biomedical Imaging, New Haven, CT USA; 2 University of Essen School of Medicine, Essen, Germany; 3 Charité University School of Medicine, Berlin, Germany; 4 Wesleyan University, Middletown, CT USA; 5 Tulane University School of Medicine, New Orleans, LA USA; 6 Ludwig Maximilian University School of Medicine, Munich, Germany; 7 University of Göttingen School of Medicine, Göttingen, Germany; 8 Ulm University School of Medicine, Ulm, Germany; 9 University of Leipzig School of Medicine, Leipzig, Germany; 10 Division of Computational Pathology, Department of Pathology & Laboratory Medicine, Indiana University School of Medicine, Indianapolis, IN USA; 11 Department of Radiology and Department of Pathology & Laboratory Medicine, Perelman School of Medicine, University of Pennsylvania, Philadelphia, PA USA; 12 School of Clinical Dentistry, University of Sheffield, Sheffield, England; 13 Diagnostic, Molecular and Interventional Radiology, Biomedical Engineering Imaging, Mount Sinai Hospital, New York City, NY USA; 14 Visage Imaging, GmbH, Berlin, Germany; 15 Visage Imaging, Inc., San Diego, CA USA; 16 Department of Therapeutic Radiology, Yale School of Medicine, New Haven, CT USA; 17 Center for Outcomes Research and Evaluation (CORE), Yale School of Medicine, New Haven, CT USA

**Keywords:** CNS cancer, Metastasis

## Abstract

Resection and whole brain radiotherapy (WBRT) are standard treatments for brain metastases (BM) but are associated with cognitive side effects. Stereotactic radiosurgery (SRS) uses a targeted approach with less side effects than WBRT. SRS requires precise identification and delineation of BM. While artificial intelligence (AI) algorithms have been developed for this, their clinical adoption is limited due to poor model performance in the clinical setting. The limitations of algorithms are often due to the quality of datasets used for training the AI network. The purpose of this study was to create a large, heterogenous, annotated BM dataset for training and validation of AI models. We present a BM dataset of 200 patients with pretreatment T1, T1 post-contrast, T2, and FLAIR MR images. The dataset includes contrast-enhancing and necrotic 3D segmentations on T1 post-contrast and peritumoral edema 3D segmentations on FLAIR. Our dataset contains 975 contrast-enhancing lesions, many of which are sub centimeter, along with clinical and imaging information. We used a streamlined approach to database-building through a PACS-integrated segmentation workflow.

## Background & Summary

Brain metastases (BM) develop in up to 30–40% of patients with a primary malignancy, particularly those with lung cancer, breast cancer, and melanoma^1,2^ Palliative treatment for BM includes resection, whole brain radiotherapy (WBRT), and, more recently, stereotactic radiosurgery (SRS)^1^ Although WBRT can reduce the neurological symptoms of BM, the overall survival has been shown to be decreased in patients with certain risk factors, including older age, lower baseline cognitive performance status, and >3 BM^3,4^ SRS provides a more targeted and less toxic approach to BM treatment than WBRT and can be performed when patients present with >10 lesions although its predominant use is still in treatment of localized metastatic disease^5,6^ In fact, one meta-analysis revealed a significant improvement in performance status and local control in patients treated with WBRT plus SRS compared to WBRT alone^7^ Localization and accurate delineation of BM margins are critical for effective SRS treatment^8^ In addition, differentiation of BM from high-grade gliomas, such as glioblastoma, can be challenging, and textural analysis of the peritumoral environment on T2/FLAIR MRI sequences can aid in differentiation of these tumor subtypes^9^

To address the challenge of BM diagnosis and delineation, several artificial intelligence (AI) tools, including machine learning (ML) and deep learning (DL) algorithms, have been developed in the past decade^8,10–14^ While many of these algorithms showed promising results in BM diagnosis and auto-segmentation, there is still a large gap in the clinical implementation and adoption of these algorithms^12,15^ One reason for this gap is the lack of algorithm generalizability to real-world datasets. In fact, many algorithms are trained and developed on small single-institution hospital datasets that lack diversity in patient populations and imaging protocols, which are often present in the clinical setting^12^ In fact, one meta-analysis of BM algorithms revealed that the average sample size of datasets used to train algorithms was around 150, with half of the studies explicitly including patients with only solitary BM^12^ Thus, there is a critical need for large, diverse, and open-access datasets to better train AI algorithms and to challenge AI models to perform accurate assessments on a large breadth of patient cases^12^ To date, there are only two publicly available BM datasets, both of which contain under 200 patients with pretreatment segmentations solely on T1 post-contrast^16,17^.

We curated a dataset of 200 patients with a clinical or pathological diagnosis of BM with accompanying clinical and qualitative/quantitative imaging information^18^ In addition to enhancing tumor 3D segmentations, our dataset also provides 3D segmentations of necrotic tumor portions on T1 post-contrast and peritumoral edema on FLAIR. Our dataset includes several sub-centimeter contrast-enhancing lesions, which are critical for training algorithms to recognize subtle lesions on imaging^18^ Manual 3D tumor segmentations using a commercially available semi-automatic segmentation tool was performed in a novel workflow directly in a research instance of our PACS (AI Accelerator, Visage Imaging, Inc., San Diego, CA)^19^ which allowed for the creation and validation of segmentations in an accelerated time frame. Our dataset is publicly available on The Cancer Imaging Archive (TCIA) platform with all tumor segmentations (contrast-enhancing, necrotic, and peritumoral edema), standard MRI sequences (T1, T1 post-contrast, T2, and FLAIR), and an Excel file containing clinical and qualitative/quantitative imaging information^18^ We hope that our dataset contributes to the training and validation of future BM AI algorithms with the goal of their implementation, translation, and adoption in clinical practice for BM diagnosis and treatment.

## Methods

### Subject characteristics

Patients were queried from the Yale New Haven Hospital (YNHH) database from 2013 to 2021, the YNHH tumor board registry in 2021, and the YNHH Gamma Knife registry from 2017 to 2021. Inclusion criteria were a clinical or pathological diagnosis of brain metastasis confirmed on the electronic medical record and availability of all four pretreatment standard MRI sequences (T1, T1 post-contrast, T2, and FLAIR) without significant motion artifact. There was a total of 200 patients included in the dataset^18^ Of the 200 patients, the following was the breakdown of primary tumor origin: non-small cell lung cancer (86, 43%), melanoma (41, 20.5%), breast cancer (26, 13%), small cell lung cancer (17, 8.5%), renal cell carcinoma (16, 8%), and gastrointestinal cancers (14, 7%).

### Image acquisition

A summary of all imaging parameters for FLAIR and T1 post-contrast images of the 200 patients can be found in Table 1. The images were obtained on 1-T (4, 2%), 1.5-T (113, 56.5%), and 3-T (83, 41.5%) MRI scanners. Scanner vendors included Siemens (158, 79%), General Electric (31, 15.5%), Philips (7, 3.5%), and Hitachi (4, 2%).

**Table 1:** Summary of imaging parameters for FLAIR and T1 post-contrast sequences.

Imaging Parameter	FLAIR	T1 post-contrast
Acquisition (n, %)	2D (193, 96.5%)	2D (32, 16%)
	3D (5, 2.5%)	3D (166, 83%)
	N/A (2, 1%)	N/A (2, 1%)
Median (range) echo time (msec)	92.0 (10.0–400.0)	3.1 (1.8–26.1)
Median (range) repetition time (msec)	9000.0 (1700.0–12000.0)	1900.0 (5.9–2619.8)
Median (range) slice thickness (mm)	5.0 (1.0–5.5)	1.0 (0.9–5.0)
Median (range) slice spacing (mm)	5.0 (0.0–7.5)	0.0 (0.0–7.0)

*N/A = not available; range = minimum to maximum.

### Segmentation procedure

**Fig. 1:**
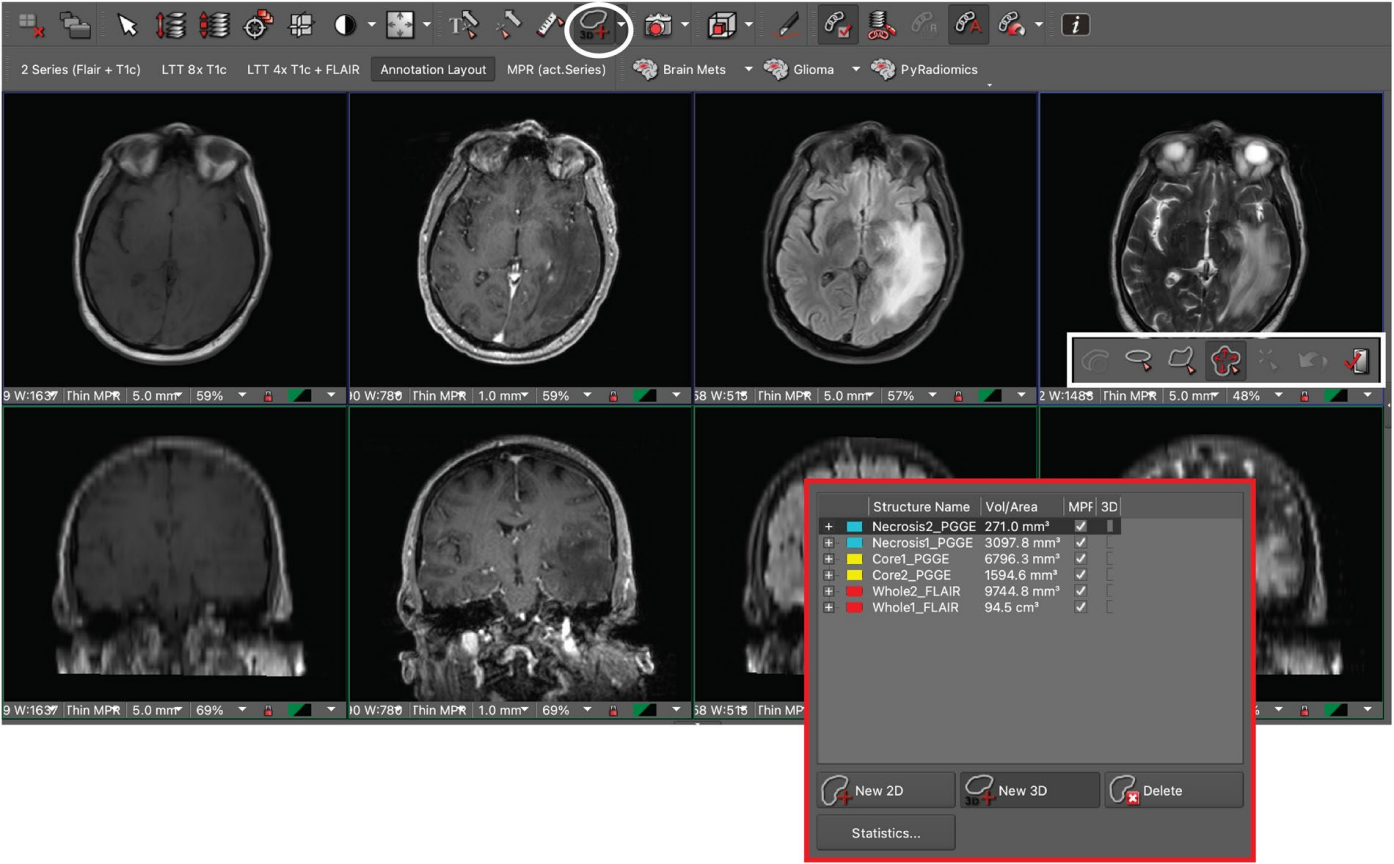
Research PACS annotation layout. The T1, T1 post-contrast, FLAIR, and T2 sequences for one patient are displayed on the eight-viewer layout after alignment with the auto-align tool. The PACS interface incorporates a 3D volumetric tool (white circle/rectangle) and displays labeled segmentations for two brain metastases in the display window (red rectangle).

The DICOM studies for all 200 patients were sent and de-identified from the clinical production (Visage 7, Visage Imaging, Inc., San Diego, CA) to a research instance of our PACS. To streamline the segmentation workflow, a custom hanging protocol and eight-viewer layout were designed to automatically 3D register and display the relevant MR imaging sequences upon study load^19,20^ Manual segmentations were performed by one medical student (L.J.) on the research PACS using a commercially available semi-automatic 3D segmentation tool as shown in Fig. 1. Research PACS annotation layout^19^.

**Fig. 2:**
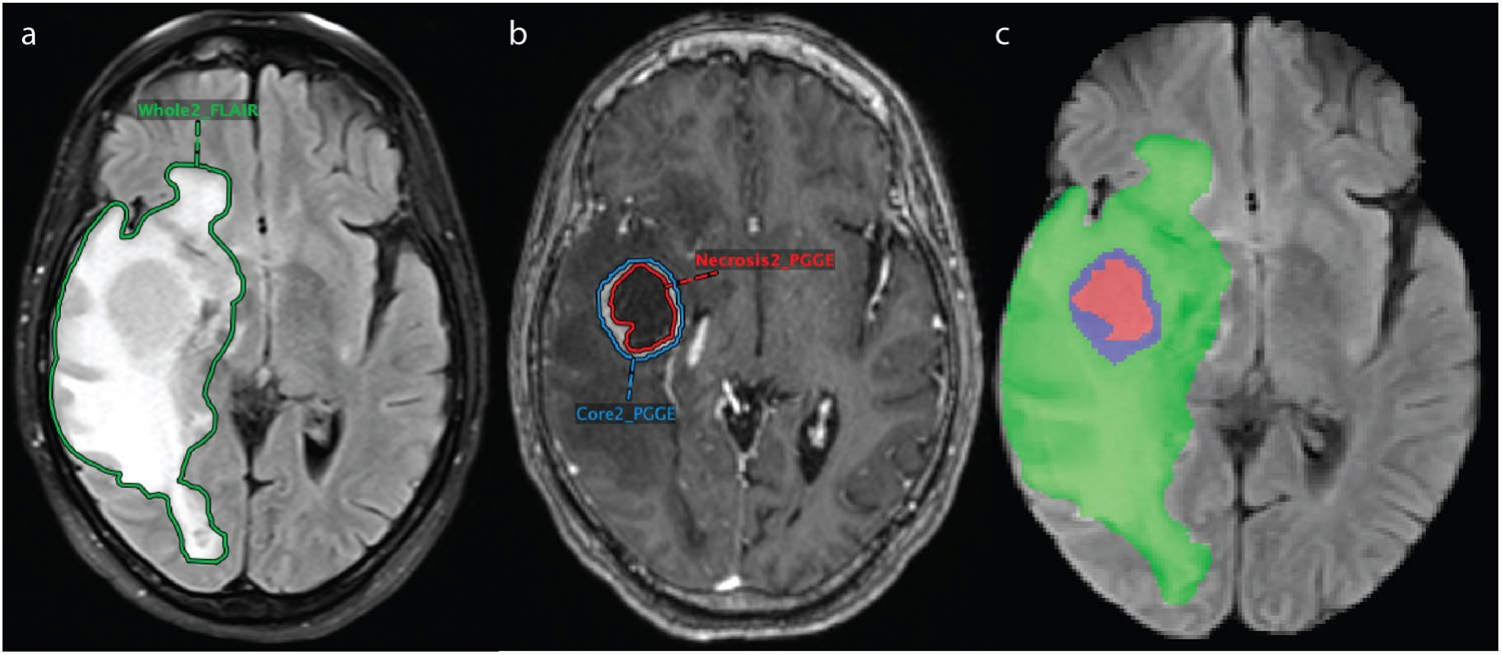
PACS-based segmentations and NIfTI masks for one patient. After auto-alignment of FLAIR and T1 post-contrast sequences, segmentation of whole tumor (“Whole2_FLAIR”), including peritumoral edema, was performed on FLAIR (**a**), and segmentations of contrast-enhancing lesion (“Core2_PGGE”) and corresponding necrotic portions (“Necrosis2_PGGE”) were performed on T1 post-contrast (**b**). (**c**) The combined segmentation masks are shown overlaid on the FLAIR sequence in NIfTI format. The green region represents peritumoral edema, the blue region represents contrast-enhancing tumor, and the red region represents necrotic tumor.

The segmentations were checked and manually revised as needed by two board-certified neuroradiologists (M.S.A. and F.M.) with more than seven years of clinical experience each. Whole tumor (including peritumoral edema) was segmented on FLAIR as shown in Fig. 2a. PACS-based segmentations of whole tumor. Whole tumor includes the entirety of the tumor, which appears hyperintense on FLAIR, and includes edema and infiltrative tissue surrounding the contrast-enhancing portion of the tumor. A total of 662 lesions had peritumoral edema surrounding contrast-enhancement. Contrast-enhancing lesions and necrotic portions were segmented on T1 post-contrast as shown in Fig. 2b. PACS-based segmentations of contrast-enhancing lesion and corresponding necrotic portions. Contrast-enhancing lesions included those that showed hyperintensity on the T1 post-contrast sequence compared to the T1 sequence. Necrotic portions included regions within contrast-enhancing lesions that were hypointense on T1 post-contrast compared to T1. These regions can also be fluid-filled and appear hyperintense on T2. In total, there were 975 contrast-enhancing lesions among all patients with 285 patients having necrotic components. Notably, because a 3D registration of the various MR imaging sequences was performed using the custom hanging protocol, the segmentation masks could be accurately copied and pasted between MR imaging sequences^19^.

### Clinical data and anonymization

Clinical data for all patients were collected from the electronic medical record. They include the following: age at diagnosis, sex, ethnicity, smoking history at diagnosis in pack-years, primary tumor origin, presence of extranodal metastasis, and time to death or last note in the electronic medical record as of July 2022. The following qualitative/quantitative imaging features were included: presence of infratentorial involvement, total number of lesions with contrast-enhancement, necrosis, and peritumoral edema, total volume of all regions (contrast-enhancing, necrotic, and peritumoral edema), ratio of necrotic to contrast-enhancing volume, and ratio of peritumoral edema to contrast-enhancing volume.

De-identification was implemented on the research server and occurred directly upon receipt of the DICOM images from either the PACS production system or the long-term archive. No non-anonymized images were stored on the research server. The de-identification removes/modifies all metadata that have identifiable information according to the DICOM standard PS3.15 2018b Appendix E “Attribute Confidentiality Profiles”. Specifically, the “Basic Profile” combined with the “Clean Descriptors Option”, the “Clean Structured Content Option” and the “Retain Longitudinal Temporal Information with Modified Dates Option” were implemented. The PatientID, Accession number, and StudyInstanceUID were removed and replaced with a computed unique ID that is calculated using hash functions and a hash key. While this process is not reversible, it does guarantee that, if another study for the same patient is sent through the pipeline later, those new objects are assigned to the same patient on the research server, unless the hash key in the pipeline is changed. Likewise, additional images/series for the same study would be assigned to the same de-identified study. The MR images and 3D segmentation masks were exported as NIfTI files from the research server using the Python Visage application program interface (API). The Cancer Imaging Phenomics Toolkit (CaPTk)^21^ and Federated Tumor Segmentation (FeTS)^22^ pipelines were used to pre-process all sequences and segmentations for each patient. The pre-processing steps included image co-registration to the SRI24 anatomical template, resampling to a uniform isotropic resolution (1 mm^3^), and skull stripping to maintain patient anonymity. Both the PACS annotation system and CaPTk toolkit used a rigid registration method for the images.

### Ethical approval

The study was conducted according to the guidelines of the Declaration of Helsinki and approved by the Institutional Review Board (or Ethics Committee) of Yale University, protocol 2000029055, approved on 10/01/2020. The IRB waived participant consent given data anonymization and approved open publication of the data.

## Data Records

The dataset has been deposited to The Cancer Imaging Archive (TCIA)^18^ Each patient has a total of five associated NIfTI files with four image files of the standard sequences (T1 pre-contrast, T1 post-contrast, T2, and FLAIR) and a fifth segmentation file with combined masks from T1 post-contrast and FLAIR segmentations. The segmentation file has three labels: Label 1 (red) represents tumor necrosis, Label 2 (green) represents peritumoral edema, and Label 3 (blue) represents contrast-enhancing tumor as shown in Fig. 2c. Combined segmentation NIfTI mask for one patient. The dataset also contains one Excel file with clinical and qualitative/quantitative imaging information^18^ The patients are labeled with anonymized identifiers.

## Technical Validation

All patients had brain metastases and primary tumor of origin confirmed either pathologically or clinically through the electronic medical record. In addition, only patients with high-quality T1, T1 post-contrast, T2, and FLAIR images without significant motion artifacts were included in the final dataset^18^ All segmentations were independently validated by two neuroradiologists (M.S.A. and F.M.) with more than seven years of clinical experience each. After exporting to NIfTI format, standard sequences and segmentation files for all patients were opened on the ITK-SNAP software. Since all segmentations were combined into one mask per patient during preprocessing, a neuroradiologist (M.S.A.) made additional adjustments to the combined segmentation mask, which involved correction of any over or under segmented regions of interest (i.e. tumor necrosis, peritumoral edema, and contrast-enhancing tumor) after opening the segmentation file on ITK-SNAP and aligning it with the standard sequences. A medical student (D.R.) double checked and adjusted the revised NIfTI segmentation masks and manually counted the number of lesions with contrast-enhancement, necrosis, and peritumoral edema for each patient.

## Usage Notes

After completion of the data upload process, the NIfTI files can be downloaded from TCIA (https://www.cancerimagingarchive.net) public collection “Pretreat-MetsToBrain-Masks” at 10.7937/6be1-r748 and opened on segmentation platforms that support NIfTI format^18^.

## Data Availability

The image pre-processing code used to build the dataset can be found at the following link: https://cbica.github.io/CaPTk/preprocessing_brats.html.
